# Ketogenic Diet-Induced Alterations in Neuronal Signaling-Related Proteins are Not Due to Differences in Synaptosome Protein Levels

**DOI:** 10.1007/s12035-025-04988-1

**Published:** 2025-04-29

**Authors:** Rachel Fletcher, Meagan Hoppe, Joseph A. McQuail, Caesar M. Hernandez, Abbi R. Hernandez

**Affiliations:** 1 University of Florida, Gainesville, FL USA; 2 Sidney Kimmel Medical College, Thomas Jefferson University, Philadelphia, PA USA; 3 Department of Pharmacology, Physiology, and Neuroscience, University of South Carolina School of Medicine, Columbia, SC USA; 4 Department of Medicine, Division of Geriatrics, University of Alabama at Birmingham, Gerontology & Palliative Care, 845 19th St. South Rm 768, Birmingham, AL 35205 USA

**Keywords:** Aging, Synapse, GABA, Glutamate, Receptor

## Abstract

Impaired cognitive function is a hallmark of advancing age, and the potential to reverse or delay these cognitive deficits through dietary intervention holds promise for improving quality of life for older adults. Specifically, ketogenic diets (KDs) have now been widely used for the treatment of several neurological and peripheral disorders, including diseases profoundly affecting cognitive health, of which advanced age is the single greatest risk factor. However, the precise mechanisms of the efficacy of KD-based interventions to reverse age-related cognitive and neurobiological declines are not fully elucidated. We have previously demonstrated that a KD improves cognitive function, with concurrent increases in global levels of proteins related to synaptic signaling in the aging hippocampus (HPC) and prefrontal cortex (PFC). Despite these advances, it remains unclear as to whether these changes reflect biochemical modifications specifically localized to synaptic terminals. To address this important, unanswered question, we purified synaptosomes from the HPC and PFC of aging rats fed a KD or control diet (CD) for a minimum of 4 months and quantified 10 proteins related to synaptic transmission. In contrast to previous studies of global protein expression, the signaling proteins measured did not show significant differences between diet groups in synaptosomes isolated from either region. When pre-mortem performance on an Object-Place Paired Association task was considered, we found a significant correlation between several proteins within the HPC and PFC synaptosomes of CD-fed rats, more pronounced in CD-fed aged rats, that are conspicuously absent in KD-fed rats from both age groups. Moreover, there is a significant alteration in the ratio of VGAT/VGluT1, markers of excitatory and inhibitory synaptic vesicles, in the PFC with dietary intervention that is absent in the HPC, confirming prior reports of regionally specific alterations in excitatory and inhibitory signaling post KD. These new and extended findings reveal important, naturally occurring associations between protein levels localized to synaptic terminals, while clarifying that effects KD likely increase synaptic abundance without altering the biochemical composition of isolated synapses.

**Supplementary Information:**

The online version contains supplementary material available at 10.1007/s12035-025-04988-1.

## Introduction

As the proportion of individuals above the age of 60 rapidly increases [1], so does the need to prevent declining cognitive function, which critically impedes independence and quality of life for older adults. The prefrontal cortex (PFC) and hippocampus (HPC) are critical in supporting higher cognitive processes [2–4] and particularly vulnerable to aging [5–11]. Thus, alterations in the function of these regions may contribute to profound impairments in cognition and serve as ideal targets for understanding the mechanisms underlying dysfunction with age.

The absence of overt declines in neuron number with advancing age [12–15] indicates that changes are likely attributed to declining neuron *function*. One possible mechanism for neuronal dysfunction with age is the loss or alteration of synaptic connections between neurons, which are vital for proper neurobiological function [12, 16, 17]. Synapse loss plays a role in impaired cognition in both nonpathological aging and Alzheimer’s Disease (AD). In fact, individuals with early AD or mild cognitive impairment have a significantly lower synapse count in the dentate gyrus relative to those with no cognitive impairment [18]. An additional, but not mutually exclusive, avenue for age-related decline in brain function is through impaired metabolic capability, as brain glucose metabolism declines with age [19–24], and may be a significant contributing factor to age-related cognitive dysfunction, as the high energy demand of the synapses is not met. Furthermore, these bioenergetic deficits may, in fact, underlie declining synaptic connections and functioning with age and AD. Unlike declining glucose-dependent metabolic processes, ketone body metabolism is not negatively affected by aging [22, 25, 26], and provides a way to partially circumvent glucose-dependent metabolic demands. Thus, nutritionally induced ketosis may ameliorate deficits caused by impaired glucose homeostasis in the aged brain.

Our previous work has shown that a 12-week ketogenic diet (KD) is variously associated with improved cognitive function [25], changes in transporter protein expression [25, 27] and the expression of genes related to inhibitory and excitatory neuronal signaling [28]. Notably, the magnitude and direction of these diet-induced changes varies across different brain regions. Intriguingly, the effects on transporter expression differed across hippocampal (HPC) and prefrontal cortical (PFC) tissues in these studies [25, 29]. Within the HPC, the KD reversed age-related reductions in VGLUT1 expression, the transporter that packages glutamate into vesicles for synaptic release [25]. In both the HPC and PFC the vesicular GABA transporter VGAT was significantly upregulated following the KD [25, 29]. However, these studies examined protein expression at a global level, leaving unclear whether the observed changes reflect a broad increase in the abundance of excitatory and inhibitory synaptic connections, or if these signaling proteins are selectively enriched within synapses of the aging brain by KD. The latter possibility could suggest an enhanced potential for neurotransmission at these synaptic sites. Similarly, it is unclear to what extent biochemical changes localized to synaptic terminals are reliably linked to cognitive performance in aging rats, regardless of diet [30, 31]. To address these important, unanswered questions, we extended our previous work by purifying synaptosomes from young and aged rats fed a KD or control diet for a minimum of 4 months and quantified several proteins related to synaptic transmission, and established associations with cognitive performance.

## Methods

### Subjects

A total of 18 young (4 months; n = 16 male, n = 2 female) and 22 aged (20 months; n = 19 male, n = 3 female) Fischer 344 × Brown Norway F1 Hybrid rats from the NIA colony at Taconic Farms were utilized in this study. Limited availability of female rats (5 total; 2 young and 3 aged) prevented the study from utilizing equal numbers of each sex. The control diet (CD) group consisted of 9 young (n = 8 male, n = 1 female) and 13 aged (n = 10 male, n = 2 female) rats, and the KD (KD) group had 9 young (n = 8 male, n = 1 female) and 9 aged (n = 8 male, n = 1 female) rats. Cognitive characterization of these subjects has been published previously [25] on an object-place paired association (OPPA) task in which rats must associated a particular object with a specific location within a continuous alternation maze. All experimental procedures were performed in accordance with the National Institutes of Health guidelines and were approved by Institutional Animal Care and Use Committees at the University of Florida.

### Diet

Rats were randomly assigned to either a ketogenic diet (KD), which is high in fat and low in carbohydrates (75.85% fat, 20.12% protein, 3.85% carbohydrate; Lab Supply; 5722 Fort Worth, Texas), mixed with MCT oil (Neobee 895, Stephan, Northfield, Illinois), or to a calorically and macronutrient matched control diet (CD; 16.35% fat, 18.76% protein, 64.89% carbohydrate; Lab Supply; 1,810,727, Fort Worth, Texas; for details on diet, see Hernandez et al., 2017). Regardless of diet group, rats were meal fed once daily. At the same time each day (approximately 15:00), rats were weighed and fed 51 kcals, which is enough to maintain a healthy weight on both the KD and CD [29]. Rats remained on these diets for 4–6 months and had ad libitum access to water.

### Confirmation of Ketosis and Tissue Isolation

To confirm the metabolic state of ketosis in KD rats, β-hydroxybutyrate (BHB; mmol; L), the prominent ketone body in the blood, and glucose levels (mg; dL) were determined using the Precision Xtra blood monitoring system (Abbott Diabetes Care, Inc., Alameda, CA, United States) at the time of sacrifice. A glucose-ketone index, calculated as $\frac{{~}^{Glucose (\frac{mg}{dL})}\!\left/ \!{~}_{18}\right.}{BHB (\frac{mmol}{L})}$, with higher values indicating a lesser state of ketosis, was calculated from levels determined on the day upon which tissue was collected (Fig. S1). Maintenance of nutritional ketosis throughout the duration of dietary implementation can be found in [25].

Rats were sacrificed with isoflurane-saturated cotton (Abbott Laboratories, Chicago, IL, United States) that was separated from the animal by a wire mesh shield in a glass bell jar. Once rats lost the righting reflex after 30 s in the jar, they were immediately euthanized by decapitation. Hippocampal (HPC) and prefrontal cortical (PFC) tissue was immediately extracted from the right hemisphere, frozen on dry ice, and stored at − 80ºC.

### Synaptic Fractionation

HPC and PFC tissue was subject to purification in order to extract synaptosomes for quantification of synaptic proteins (for more details about synaptic fractionation protocol, see Dunkley et al., [32]). Briefly, a microinfusion pump (Harvard Apparatus, MA, USA) was used to fill 12-mL polycarbonate centrifuge tubes with four layers of discontinuous Percoll gradients (23%, 15%, 10%, and 3%). Pumps loaded the gradients at a speed of 0.5 mL/min in order to prevent disturbance of gradient layers. HPC tissue was fully homogenized in ice-cold homogenizing buffer and centrifuged at 3,600 rpm for 10 min at 4ºC. The supernatant was collected then pipetted carefully on top of the 3% Percoll layer in each tube. Gradients were centrifuged for 20,000 rpm at 4ºC for 5 min with no brake. Following centrifugation, the supernatant separated into fractions based on density and the synaptic fraction (both bands between the 23% and 10% gradients) was collected from each tube. Synaptic fractions were diluted in ice-cold sucrose/EDTA buffer, centrifuged at 16,000 rpm for 30 min at 4ºC. Finally, 200 μL of the synaptic fractions were collected for use in western blotting. Fractions were confirmed via cryo electron microscopy (Fig. S2).

### Western Blotting

For full details of our methods, see previous studies from our group [8, 27, 29, 33]. Briefly, each western blot was conducted in triplicate, with the order of samples loaded into lanes randomized between gels. A total of 5 µg of protein (quantified via BCA assay) was run on 4–15% TGX gels (Bio-Rad Laboratories, Hercules, CA, United States) in tris–glycine running buffer (Bio-Rad) and transferred to a 0.45 µm pore nitrocellulose membrane. Li-Cor Revert total protein stain was utilized according to manufacturer instructions prior to blocking and overnight incubation in primary antibody solution (see Table 1 for antibody details). Secondary antibodies were incubated for 1 h at room temperature before scanning at 685 and 785 nm lasers on the Odyssey IR Scanner. This process was repeated with an additional primary and secondary antibody. See Fig. S3 for full sized blots.

**Table 1:** Primary and Secondary Antibodies for all experiments

PROTEIN TARGET	COMPANY	PART NUMBER	LOT NUMBER	HOST	MOLECULAR WEIGHT	DILUTION FACTOR
GABA(B)R1 A & B	Cell Signaling Technology	3835	1	Rabbit	100 & 130	1:1,000
VGLUT1	Millipore	MAB5502	3215491; 3232354	Mouse	60	1:1,000
VGAT	Millipore	AB5062P	3200577; 3243407; 3253341	Rabbit	57	1:1,000
GABA(B)R2	Cell Signaling Technology	3839	1	Rabbit	105	1:1,000
NR2A	Millipore	05 - 901R	3091636	Rabbit	170	1:5,000
NR2B	Millipore	05–920	3117749	Mouse	180	1:2,000
MGLUR2	Abcam	ab15672	GR300119 - 24; GR300119 - 25; GR300119 - 26	Mouse	99	1:2,000
DONKEY (RED)	Li-Cor	926–68022	40416–01	Anti-mouse	-	1:15,000
DONKEY (RED)	Li-Cor	926–68028	C40611 - 01	Anti-chicken	-	1:15,000
DONKEY (RED)	Li-Cor	926–68024	C40610 - 08	Anti- goat	-	1:15,000
DONKEY (GREEN)	Li-Cor	926–32213	C90129 - 05	Anti-rabbit	-	1:20,000

### Statistical Analysis

For all protein expression results, data was first normalized to total protein staining within each lane, and then expression was normalized to the average of all young rats within the CD group. Data were analyzed using a two-factor ANOVA with the between subjects factors of age (2 levels: young and aged) and diet (2 levels: CD and KD). Normality was checked via D’Agostino & Pearson testing and all values were > 0.05 (passed normality testing). Outlier analyses were run on all datasets, and all statistical analyses presented herein were run with and without female subjects (data presented herein includes females) to ensure they were not outliers, though true effects of sex are not able to be performed with such low female availability. All analyses were performed with GraphPad Prism v9.5.1 or R v4.1.2. Statistical significance was considered at p-values less than 0.05. Correlation analysis was performed using RStudio with the rcorr function of the Hmisc package [34] and plotted using the package corrplot [35].

## Results

### GABAergic Proteins

**Fig. 1:**
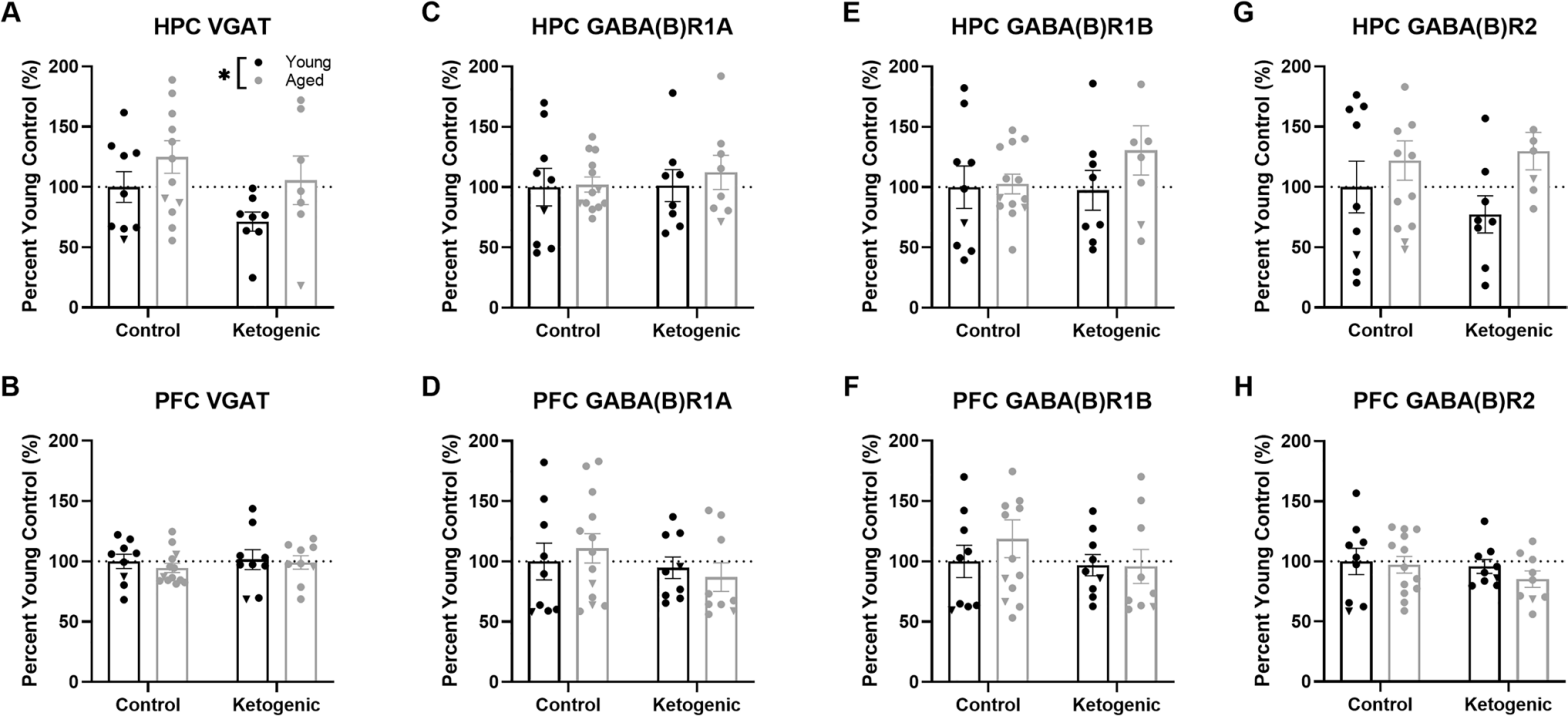
GABAergic signaling related protein expression within hippocampal (HPC) and prefrontal cortical (PFC) synaptosome isolates. (**A**) There was a significant main effect of age on HPC VGAT expression (p = 0.05), (**B-H**) but no other effects of age or diet on any other GABAergic signaling related proteins within the HPC or PFC (p ≥ 0.05 for all comparisons). Data are represented as group means ± 1 SEM with individual dots representing each biological replicate (black = young, gray = aged, triangle = female), * = p < 0.05

GABAergic signaling-related proteins were quantified within HPC and PFC tissue: VGAT, GABA(B)R1A, GABA(B)R1B and GABA(B)R2 (Fig. 1). Within the HPC, there was a significant effect of age on VGAT expression (F_[1, 33]_ = 4.14; p = 0.05) such that aged rats had significantly elevated VGAT relative to young, regardless of diet (effect of diet: F_[1, 33]_ = 2.74; p = 0.11; interaction with age: F_[1, 33]_ = 0.11; p = 0.75). Similarly, there was a trend towards an effect of age on GABA(B)R2 expression (F_[1, 33]_ = 4.08; p = 0.052) such that aged rats had increased expression relative to young, without any influence of diet (F_[1, 33]_ = 0.16; p = 0.69; interaction: F_[1, 33]_ = 0.69; p = 0.41). For the remaining proteins within the HPC and for all four within the PFC, there were no significant effects of age (p > 0.25 for all comparisons), diet (p > 0.26 for all comparisons) and there were no interactions between these two variables (p > 0.32 for all comparisons).

### Glutamatergic Proteins

**Fig. 2:**
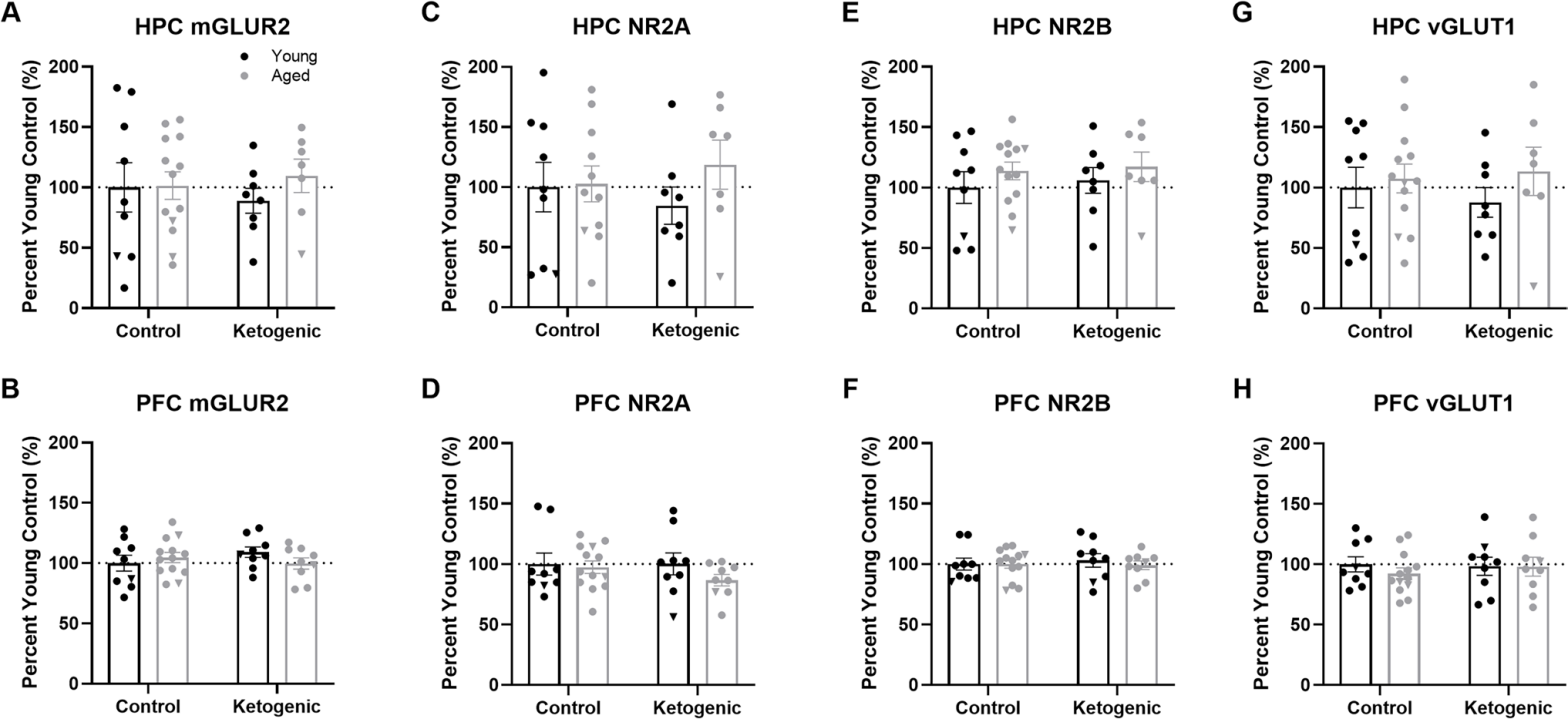
Glutamatergic signaling related protein expression within hippocampal (HPC) and prefrontal cortical (PFC) synaptosome isolates. (**A-H**) There were no significant effects in either of the brain regions of age (p > 0.25 for all comparisons), diet (p > 0.46 for all comparisons) or the interaction between diet and age (p > 0.17 for all comparisons) on the glutamatergic signaling-related proteins. Data are represented as group means ± 1 SEM with individual dots representing each biological replicate (black = young, gray = aged, triangle = female)

Glutamatergic signaling-related proteins were quantified within HPC and PFC tissue: mGluR22, NR2A, NR2B and vGluT1 (Fig. 2). Within both brain regions, there were no significant effects of age (p > 0.25 for all comparisons), diet (p > 0.46 for all comparisons) and there were no interactions between these two variables (p > 0.17 for all comparisons) for all glutamatergic signaling-related proteins quantified.

### Correlation with Behavioral Performance on Object Place Paired Association

A correlation matrix was conducted for each age, diet and brain region, using the Hmisc and corrplot R packages [34, 35], for each protein and OPPA performance on days 12–15. This timepoint was chosen as this is a timepoint at which diet-induced improvements in behavioral performance have been observed [25]. Notably, there was a significant effect of age on task performance, with young rats outperforming their aged counterparts (see [25] for additional details regarding cognitive characterization of these subjects).

**Fig. 3:**
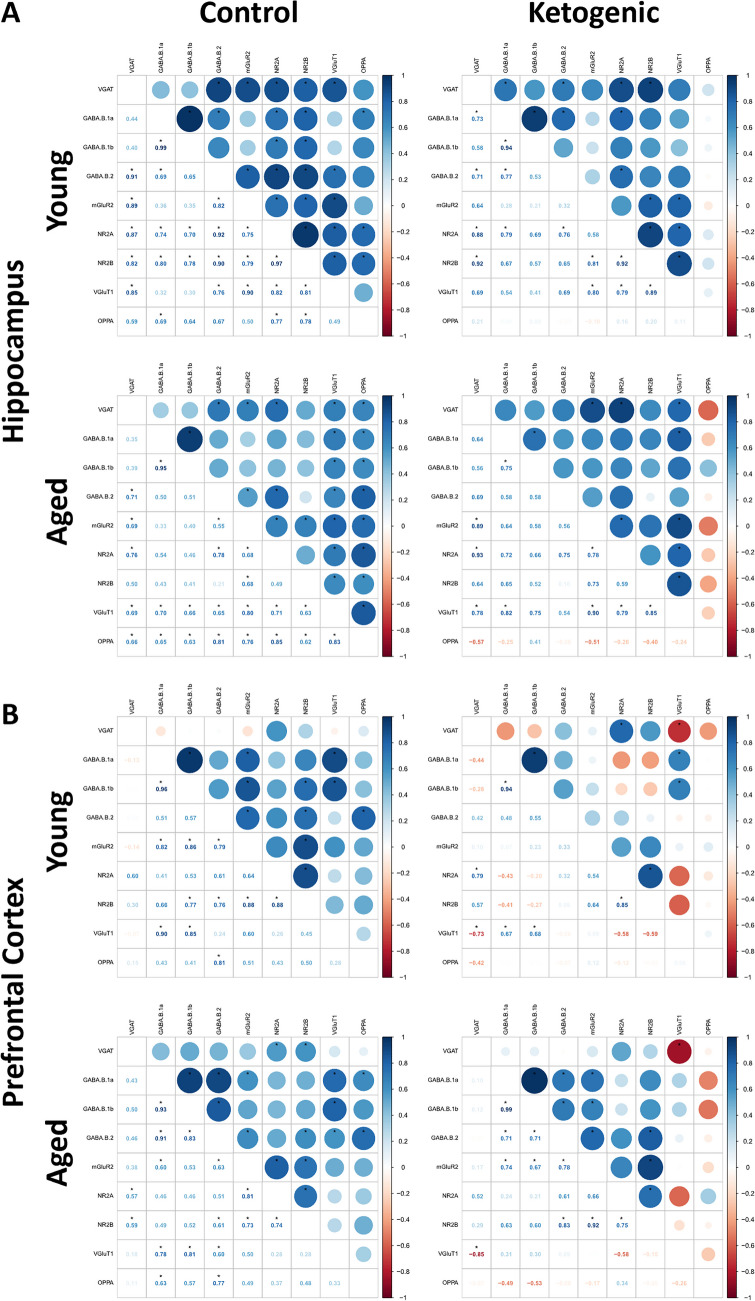
Correlation matrix for (**A**) hippocampal and (**B**) prefrontal cortical synaptosomal protein concentrations with each other and with object-place paired association (OPPA) task performance in young and aged rats on the control (left) and ketogenic (right) diets. Values in the lower left quadrant indicate the correlation coefficient, whereas values in the upper right quadrant indicate correlation direction (color coded using scale shown on the right) and strength (indicated by circle size), * indicates p < 0.05

Several proteins correlated with behavioral performance in control-fed rats of both ages, but the same was not true of ketogenic-fed subjects. Increased HPC levels of the excitatory proteins mGluR2, NR2A, NR2B and VGLUT1 and the inhibitory proteins GABA(B)R1a, GABA(B)R1b, GABA(B)R2, and VGAT all significantly correlated with improved performance in aged rats fed the CD (p < 0.01 for all comparisons; Fig. 3A). However, aged rats fed the KD did not demonstrate this same trend, as none of these excitatory or inhibitory proteins significantly correlated with behavioral performance in this group (p ≥ 0.17 for all comparisons). A similar trend was observed within the PFC from aged rats fed the CD for a small number of proteins, including GABA(B)R2 (p = 0.003) and GABA(B)R1a (p = 0.03; Fig. 3B), with no significant correlations within PFC from aged rats fed the KD (p ≥ 0.14 for all comparisons). Thus, these data may mean that age-related decrements in protein levels that contribute to poorer cognitive outcomes are reduced as KD ameliorates protein expression and restores cognitive performance.

Similar trends were observed in young rats, though for fewer proteins. Higher levels of HPC GABA(B)1a, NR2A and vGLUT1 (p < 0.04 for all comparisons), as well as PFC GABA(B)R2 (p = 0.008) proteins corelated with better performance in young control-fed rats, though none were significantly correlated in ketogenic-fed young rats in either region (p ≥ 0.25 for all comparisons). Again, these data may indicate that a KD is capable of enhancing protein expression files where lower expression limits cognitive performance in traditionally fed subjects.

In addition to correlations with behavioral performance, correlations between proteins were investigated. While many proteins in both categories correlated with other proteins within the same category (i.e., excitatory proteins correlated with other excitatory proteins), there were some other notable relationships. Firstly, within all groups of rats, one or more excitatory proteins positively correlated with at least one inhibitory protein regardless of age and diet group. For example, mGluR2 positively correlated with GABABR2 in all control-fed rats, regardless of age or brain region and NR2A positively correlated with VGAT in all KD-fed rats in at least one brain region. Secondly, and perhaps more interestingly, within the PFC synaptosomes from both young and aged rats fed the KD, there was an inverse relationship between VGLUT1 and VGAT, the vesicular packaging proteins for glutamate and GABA respectively. However, there was no such relationship in HPC synaptosomes. In fact, this relationship was significantly positively correlated in HPC synaptosomes from aged rats on either diet, as well as in young rats on the CD.

## Discussion

Ketogenic diets (KDs) are capable of changing both gene [28, 36–39] and protein [25, 29, 40–42] levels of signaling-related molecules within the central nervous system. Moreover, they can improve cognitive function, though whether this is due to these changes in gene and protein expression or other synergistic improvements in CNS function remains unclear. Our lab previously demonstrated changes in specific signaling-related proteins following 12 weeks of a well-controlled KD in rats relative to their control-fed counterparts in an age- and brain region-specific manner [25, 29]. However, this work did not differentiate whether these changes were due to differences in content *per* synapse or due to a change in the quantity of synapses themselves. Moreover, this previous assessment did not investigate how any changes in protein were related to cognitive performance, which differs among individuals even within age groups or diet treatments. Therefore, this work expanded upon our previous study by isolating synaptosomes from hippocampal (HPC) and prefrontal cortical (PFC) tissue from rats fed a KD or control diet (CD) for a minimum of 4 months. Our data demonstrate that synaptosomal protein levels of 8 different signaling-related proteins did not significantly differ in either region following dietary intervention, suggesting previous results are indicative of alterations in synaptic abundance rather than biochemical modifications at the level of the synapse. Secondary to observations of diet effects, we discovered a reliable age-dependent increase in synaptic VGAT in the HPC. This finding is significant as previous work implicates a loss of interneurons and inhibitory terminals throughout the aged hippocampus. Therefore, these findings provide evidence for biochemical compensations at surviving inhibitory synapses.

This work also expands upon previous studies by investigating correlations between synaptosome protein expression and cognitive performance on an object-place paired association (OPPA) task. There is a reliable age-related impairment in the ability to perform these types of tasks [25, 43], as well as differences in neuronal firing across PFC and HPC subregions across age [28, 44, 45]. Our data indicate that HPC synaptosome mGluR2, NR2A, NR2B, VGLUT1, GABA(B)R1a, GABA(B)R1b, GABA(B)R2, and VGAT protein quantity in aged CD rats positively correlates with the ability to perform this task, but there was no such correlation in aged KD fed rats. Because aged rats fed a KD outperform age-matched CD fed rats, the range of behavioral performance in the KD group is compressed, which reduces the strength of protein-behavior correlations in KD-fed rats.

Notably, far fewer correlations between these proteins and cognitive performance were observed in PFC synaptosomes, despite both regions being necessary for task performance [46–48]. In fact, only GABA(B)R2 and GABA(B)R1a synaptosomal protein correlated positively with cognitive performance in aged CD rats. Again, no relationships reached significance in the PFC of KD fed rats.

While expression of many individual synaptosome proteins were *positively* correlated (see Fig. 3), the association between VGLUT1 and VGAT, vesicular transporters responsible for packaging glutamate and GABA, respectively, for synapse release notably departed from this trend in KD-fed rats. Within the PFC, there is a significant, *inverse* relationship between these two proteins only when fed the KD. However, this relationship is not significant in the CD group, suggestive of an imbalance in the synaptic proteins that are essential for maintaining an optimal balance of excitatory/inhibitory (E/I) neurotransmission in this region with age [7, 49, 50]. While E/I imbalance is notable within both PFC and HPC of aged subjects, the HPC is biased towards increased activity [50–53] while the PFC is more mixed in directionality and degree of altered signaling [54–57]. In our prior work investigating the level of these proteins in the entire membrane bound fractions in the PFC, VGAT expression significantly increased and VGLUT1 expression remained unchanged, regardless of age group, after a KD [25]. Together, along with a large body of literature demonstrating alterations in GABAergic related molecules post KD [58–62], these data indicate this inverse relationship within the PFC is likely due to alterations in VGAT. Interestingly, this relationship is positive within both young and aged HPC, further demonstrating regional specificity in E/I balance across the lifespan. However, age-related impairments in metabolic resources may account for the enhanced relationship observed between protein availability and cognitive performance in aged rats relative to young, as age-related fluctuations in blood glucose are inversely related to synaptic proteins within the HPC [63]. Aged rats fed a KD may, at least in part, alleviate cognitive impairments through increased bioavailability of ATP [64, 65], restoring a biological homeostasis that was not yet disrupted in the young group.

While studies investigating the influence of KDs on synapse function and morphology are limited, our data agree with available evidence that these diets modify synapse number and morphology. Previous work utilizing a similar medium chain triglyceride (MCT)-based KD demonstrated enlargement of the average synaptic area within the CA1 of aged rats, however, these results were not generalizable to the entire brain as no such change was observed within the cerebellum [66, 67]. Moreover, a KD can increase the number of fibers projecting to the striatum and through the fimbria-fornix [68].

KD-derived alterations in synapse number and morphology deserve more attention in future studies. Firstly, one limitation of this approach is the potential loss or dilution of subregional effects, as aging-related changes in the hippocampus are non-uniform across the DG, CA3, and CA1 subregions. While electron microscopy confirms the structural preservation of axo-spinous synapses in older rats within the stratum radiatum (SR) and stratum lacunosum-moleculare (SLM) [69–72], functional declines are observed, including the suppression of KCl-evoked glutamate release in the CA3 region [73] and a reduction in the amplitude of EPSPs induced by perforant path stimulation in the DG and by Schaffer collaterals in CA1 pyramidal neurons [74–76]. In relation to our current focus on markers of glutamate and GABA synapses, a separate study in similarly aged FBNF1 rats assessed for spatial cognition, but not fed specialized diets, found a localized reduction of VGluT1 in the dorsal CA3-SLM of older rats with impaired cognition, alongside an augmentation of VGAT across several synaptic layers of CA3 and CA1 in older rats with intact cognitive function [77]. These age-related effects may interact with the ketogenic diet, as our previous gene expression analysis—parsed by subregion—indicated a significant main effect of diet on the regulation of mRNA transcripts for effectors of glutamatergic and GABAergic signaling in the DG [39]. However, gene expression alone is insufficient to estimate biochemical or physiological effects specific to synaptic compartments. Due to the need to prepare synaptosomes in sufficient abundance for protein analysis, we were unable to dissect hippocampal subregions and instead analyzed the hippocampus as a whole. Future studies may address this limitation by examining the physiological effects at synapses within the CA3 to better determine the relevance of aging and diet interactions on neurotransmission.

Secondly, although these data do replicate age-related increases in hippocampal VGAT [29], all the rats in this study were meal fed once daily and not provided food ad libitum, which may account for the lack of age-related changes in other molecules investigated herein. While this was not a caloric restriction study, all rats were slightly calorically restricted due to the nature of this feeding paradigm, as ad libitum-fed rats typically overconsume [78] and caloric restriction can eliminate age-related declines in receptor subunits despite stable synapse number [72, 79]. Finally, another limitation of the study is the imbalanced representation of biological sexes, as we focused primarily on neurobiological data from male rodents. While a parallel study of both male and female subjects would have been ideal, we were unable to obtain age-matched females from the same source, as hybrid female rats were not widely available from the National Institute on Aging during the time period when the diet study was performed. Epidemiological data indicate that the prevalence of Type 2 diabetes mellitus (T2DM), which increases with age, is greater in men than in women [80], thus enhancing the public health relevance of this study in male subjects. However, understanding sex differences in T2DM prevalence requires studies in both sexes. Although our data from male rodents are robust and consistent with previously published studies implicating age-related changes in glucose regulation and hippocampal synapses [63], there remains a need to examine the effects of the ketogenic diet and aging in female subjects to determine potential sex differences over the lifespan. This would help assess the appropriateness and effectiveness of dietary interventions in both women and men.

While the number of synapses remains stable across the lifespan within some brain regions [79], but not others [81], alterations in synapse number following a KD-based intervention may serve as a compensatory mechanism for the declining neurobiological function that occurs with age. Preservation or enhancement of synaptic morphology may be at least partially responsible for the amelioration of age-related cognitive decline observed following a KD. Thus, future work should not only include the direct quantification of synapse number, but also incorporate alterations in synapse morphology where possible. Despite the lack of significant diet-induced differences in the majority of the signaling related proteins specifically within the synaptic fraction, there is ample evidence that KDs are able to alter neurotransmission, demonstrating the usefulness of the data presented herein when continuing to investigate the mechanisms by which these effects take place.

## Supplementary Information

Below is the link to the electronic supplementary material.

Supplementary file1 (DOCX 2192 KB)

## Data Availability

The datasets used and/or analyzed during the current study are available from the corresponding author on reasonable request.
