# Supplementary material for: Ketogenic Diet-Induced Alterations in Neuronal Signaling-Related Proteins are Not Due to Differences in Synaptosome Protein Levels
Source: Mol Neurobiol. 2025 Apr 29;62(9):11632–42. doi: 10.1007/s12035-025-04988-1 (PMC12367950; doi:10.1007/s12035-025-04988-1)
Supplement: Supplementary file 1 — Supplementary file1 (DOCX 2192 KB) [file 12035_2025_4988_MOESM1_ESM.docx]

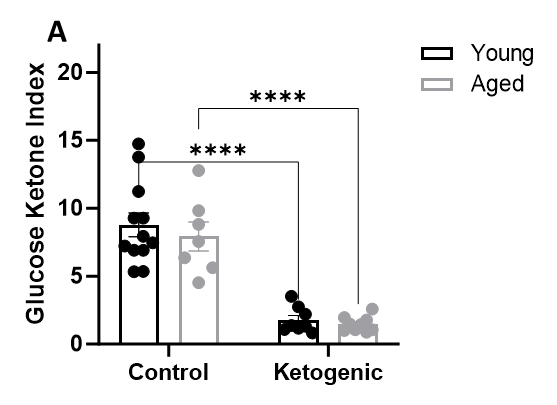
**Supplementary Material:**

**
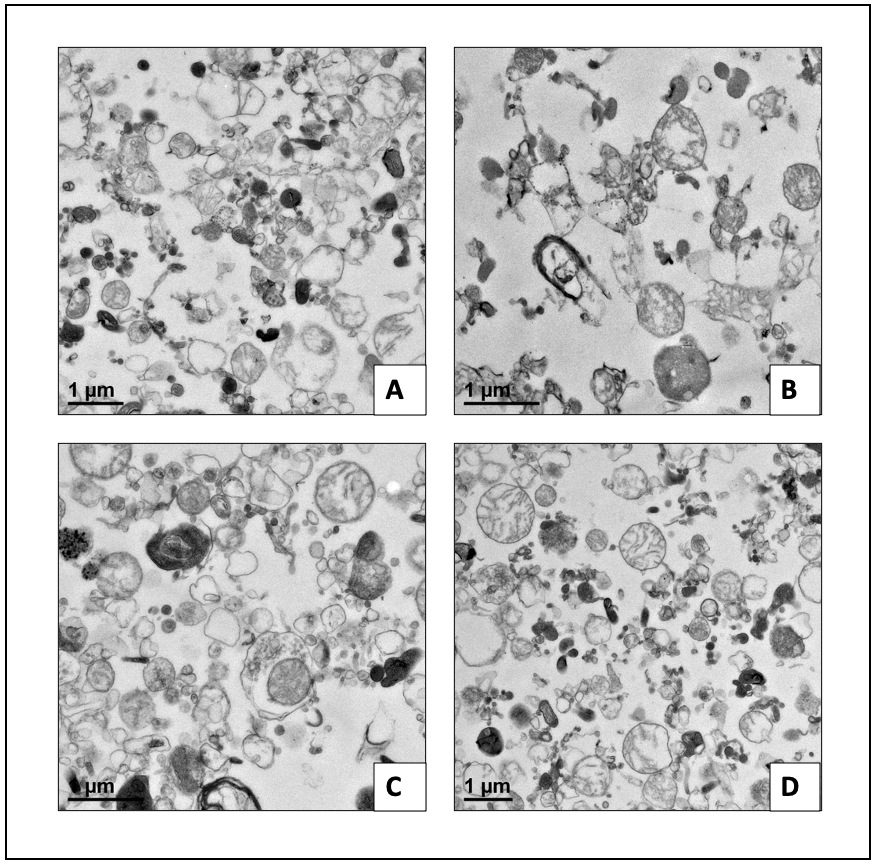
Figure S1:** (A) Confirmation of nutritional ketosis in ketogenic, but not control, rats at time of sacrifice via glucose-ketone index. Data are represented as group means ± 1 SEM with individual dots representing each biological replicate (black = young, gray = aged), **** = p < 0.0001.

**Figure S2:** Cryo-electron microscopy images for representative synaptic fractions demonstrating fractions were enriched for the synaptic plasma membrane fraction components. Regardless of age or diet, fractionation was successful in (A) young control, (B) young ketogenic, (C) aged control and (D) aged ketogenic-fed rats.


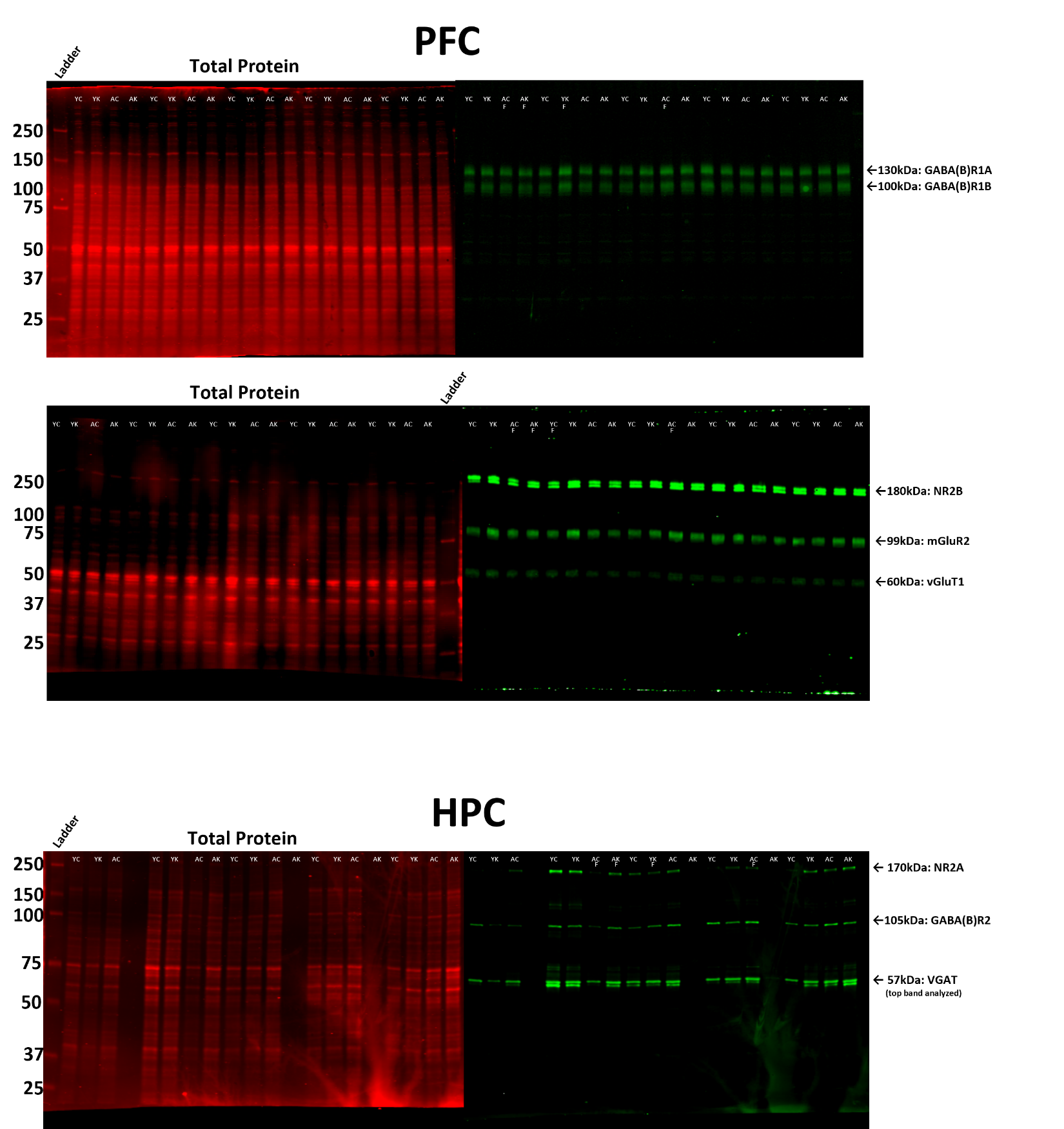


**Figure S3:** Full blots depicting each antibody used. Left/red blots depict the total protein stain (Li-Cor Revert total protein stain, imaged at 785 nm on the Odyssey IR Scanner) and right/green blots depict detection of secondary antibodies, imaged at 685 nm on the Odyssey IR Scanner. Top two example blots are from gels run with samples collected from the PFC samples and bottom blot depicts gels with HPC samples (to provide examples of each). Y= young, A= aged, C= control diet and K = ketogenic diet. Lanes containing protein from female subjects are denoted with “F”. Note: the only detected effect was in vGAT in HPC tissue, which is depicted herein.
